# Barriers to Accessing Pediatric Healthcare in Latin America: A Scoping Review

**DOI:** 10.1007/s40615-025-02510-w

**Published:** 2025-07-28

**Authors:** Laura María Gómez Trujillo, Laura Katherine Guio Cruz, Juan Sebastián Criales Laguado, Erwin Hernando Hernández Rincón

**Affiliations:** 1 Primary Care Physician, Universidad de La Sabana, Chía, Colombia; 2 Department of Family Medicine and Public Health, Universidad de La Sabana, Universitario Puente del Común, 7 Autopista Norte, Chía, Colombia

**Keywords:** Barriers to healthcare services, Child health, Primary healthcare, Child development, Pediatrics, Telemedicine, Healthcare programs

## Abstract

**Background:**

Latin America faces multiple barriers to healthcare access, particularly affecting the pediatric population. Factors such as socioeconomic vulnerability, armed conflicts, disasters, and insufficient healthcare spending contribute to these difficulties. The barriers include financial, bureaucratic, and geographical issues, which negatively impact the well-being and development of children.

**Objective:**

To identify the barriers to healthcare access in the vulnerable pediatric population of Latin America and recognize effective strategies to overcome these barriers.

**Methods:**

A scoping review was conducted in December 2024. A literature search was performed in databases such as BIREME, PubMed, and Scopus, including studies published between 2014 and 2024 in English and Spanish. Specific inclusion and exclusion criteria were applied, and the RAYYAN tool was used to manage the references.

**Results:**

Twenty-eight articles met the selection criteria. Of these, 28.57% discussed sociodemographic barriers, highlighting geographical issues and gaps in government support; other articles addressed vaccination, mainly social stigmas and a lack of supplies; and 17.8% focused on specific diseases, highlighting the lack of early access and complications. Additionally, 17.8% discussed strategies such as telemedicine to improve healthcare access in remote areas.

**Conclusion:**

The barriers to healthcare access in the pediatric population of Latin America are diverse and complex. Telemedicine has emerged as a promising strategy, ensuring access, follow-up, and medical consultation and reducing secondary complications due to late or nonexistent care for remote communities. Additional studies with robust methodologies are needed to improve the applicability of the recommendations.

## Background

Latin America is characterized by having multiple rural areas with high vulnerability risk. According to the risk management index (INFORM), the countries with the highest levels of socioeconomic vulnerability are Guatemala, Haiti, and Honduras [1]. The region’s healthcare expenditure has represented 6.64% of its gross domestic product (GDP) over the past 20 years, a much lower figure than the median of 8.97% reported in the countries of the Organization for Economic Cooperation and Development (OECD) [2]. Additionally, it is estimated that in 15 countries, around 29.3% of the population has unmet healthcare needs, equivalent to approximately 295 million people [3].

The discussing of health vulnerability involves considering multiple factors that create barriers to accessing healthcare. The main barriers include financial barriers, due to the lack of resources to pay for consultations or treatments; bureaucratic barriers, due to coverage or treatment authorization issues; and geographical barriers, due to distance and poor road infrastructure [4]. These factors constitute obstacles that prevent vulnerable populations from obtaining the medical care they require, especially the pediatric population, which has been significantly affected for a considerable time [5].

According to the Pan American Health Organization (PAHO), approximately one-third of people in Latin America (29.3%) report not seeking healthcare when needed due to multiple access barriers [5]. These barriers disproportionately impact caregivers and, consequently, the dependent pediatric population. The lack of timely and adequate care not only affects their immediate well-being but also has long-term repercussions on their physical, emotional, and cognitive development [6].

Furthermore, the pediatric population is especially vulnerable, understood as the inability to effectively resist or cope with threatening phenomena [7]. In Latin America, this situation is exacerbated by obstacles to accessing healthcare services and worsened by poverty and inequality. According to UNICEF [8], 81 million children in the region live in poverty, and total family incomes are insufficient to meet basic needs. Additionally, regional reports indicate that factors such as the lack of rural healthcare infrastructure, the shortage of healthcare professionals, and the direct and indirect costs of medical care significantly contribute to this issue [8].

Importantly, healthcare access is universal, implying that all individuals and communities should have access without any discrimination [8]. This review is relevant because it identifies the barriers to accessing healthcare in vulnerable pediatric populations in Latin America. It also aims to identify effective strategies that have been proven to overcome these barriers and strive for equal healthcare access, highlighting the use of telemedicine. This tool is utilized in teleconsultation, telepractice, and research, and it is used by specialists and other healthcare actors [9]. In this context, the study aims to identify research conducted in the last 10 years on the barriers to accessing various healthcare services faced by the pediatric population in Latin America and strategies that have reduced this vulnerability through primary healthcare over the years.

## Methods

An exploratory systematic review was conducted in December 2024 via using the methodology proposed by Arksey and O’Malley [8] and reported according to the Preferred Reporting Items for Systematic Reviews and Meta-Analyses Extension for Scoping Reviews (PRISMA-ScR) [10]. The review protocol was register in PROSPERO prior to study initiation (register number: 1060006). The process employed for this review is explained below.

### Search Strategy and Identification of Relevant Studies

The search for articles began with the selection of Medical Subject Heading (MeSH) and Health Science Descriptor (DeC) terms such as Barriers to Healthcare Services; Child Health; Primary Healthcare; Child Development; Pediatrics, Telemedicine, healthcare programs, previously defined to answer the exploratory research question, generating different search strategies.

The literature search was conducted in virtual databases such as BIREME, PubMed, and Scopus. Filters were applied during the process to limit articles written in English and Spanish to those published between 2014 and 2024. Table 1 summarizes the search strategies and filters applied.

**Table 1:** Search strategy used in every database and the applied filters

Database	Search strategy	Filters used
PubMed	(Barriers of access) AND (primary care) AND (pediatric health) AND (Latinoamerica)	Time frame: 2014 to 2024 Language: Spanish and English
BIREME	(Barriers to health access) OR (Barriers to health services access) AND (Child health) AND (Latin America) OR (America)	Time frame: 2014 to 2024 Language: Spanish and English
Scopus	(barriers AND access AND latin AND america AND health)	Time frame: 2014 to 2024 Language: Spanish and English

### Study Selection

**Fig. 1:**
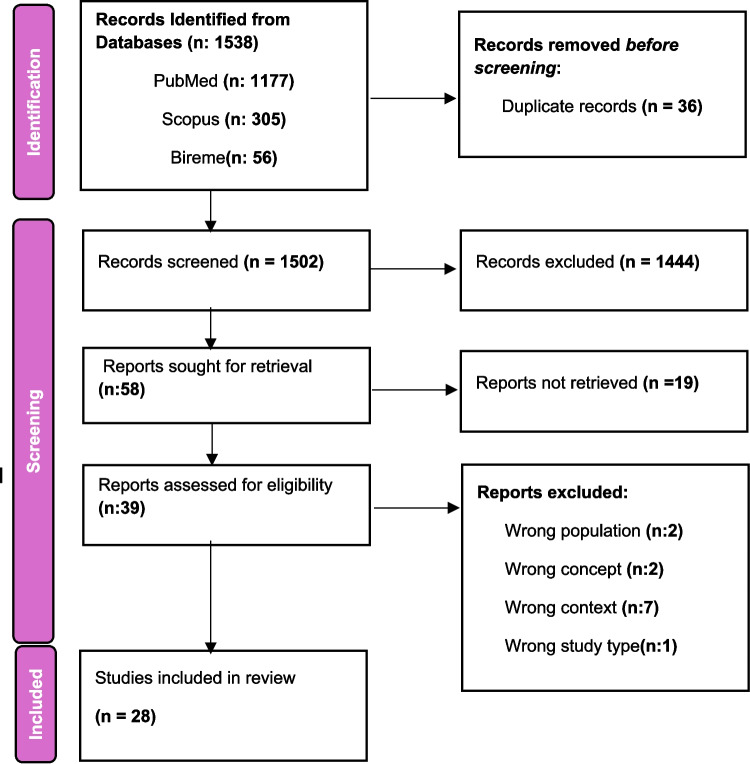
PRISMA flow diagram

After the literature search was conducted, all the references were filtered and managed through the virtual tool RAYYAN [4]. Using this tool and considering the search elements, the study selection was carried out in several steps. Initially, two independent reviewers (LMGT and JSCL) conducted the study selection through a three-stage process: screening titles, reviewing abstracts, and assessing full-text articles. The first step was to remove the duplicated records Afterwards, an initial screening was conducted on the basis solely of the titles and abstracts of the studies by two of the reviewers; any discrepancies between reviewers were resolved through discussion and consensus. If agreement was not reached, a third reviewer (LKGC) was consulted to make a final decision. Third, based on the selection criteria, a second screening was performed with a full-text reading to evaluate the relevance of the articles and their alignment with the proposed objective.The selection process was documented following the PRISMA-ScR flow diagram. Further details on the study selection process can be found in Fig. 1.

### Inclusion and Exclusion Criteria

The selection criteria were established via the population-concept-context (PCC) strategy proposed by the Joanna Briggs Institute [7]. Studies focused on vulnerable pediatric populations in Latin America were included, as well as studies mentioning the use of strategies that have proven effective in overcoming healthcare access barriers in vulnerable regions or those that may present healthcare access barriers. Studies involving pregnant women and/or populations over 18 years old were excluded, as were as case reports, unpublished theses and academic works, and studies related to major cities or high-income countries. Afterwards, the article was registered in any repository called PROSPERO.

### Data Extraction and Tabulation

Data extraction was performed via a tabulation form, which was constantly adjusted to ensure the quality of the information. Researchers defined variables to collect relevant data from each article included in this study. The extraction and tabulation processes were carried out iteratively.

### Data Collection, Summary, and Reporting of Results

Finally, the extracted information was analyzed during data tabulation and organized into relevant subcategories based on the frequency of certain thematic axes such as sociodemographic barriers, vaccination barriers, barriers to specific diseases, and strategies to overcome them, as these were the most frequent in the analyzed studies. The results are summarized and presented in tables and diagrams, as shown in Tables 2 and 3.

**Table 2:** Narrative synthesis of results organized by subcategories. Own elaboration

**Title**	**Authors**	**Year**	**Country**	Type of study	
Sociodemographic barriers (*n*=8)					
Absenteeism in child health services: a systematic review	Machado et al. [11]	2023	Brazil	Systematic review	A systematic review across 19 databases identified that over 200 million children in developing countries do not reach their potential due to risk factors. Absenteeism from appointments is high (69.4%), influenced by cultural, economic, and organizational barriers. Health education and respectful care improve adherence to child health services
Access to public health services in Latin America, A systematic literature review 2012–2022	Azañedo et al. [12]	2022	Peru	Systematic review	The health system in Latin America faces challenges due to budget constraints and social inequalities. Inadequate geographical distribution and lack of resources limit equitable access, especially in rural areas. Bureaucracy and lack of funding exacerbate the situation, affecting adolescents and vulnerable communities
Adolescent males in the City of Buenos Aires: gender-based barriers to health care and prevention	Tajer et al. [13]	2019	Argentina	Qualitative study	The research addresses the difficulty of including the experiences and needs of adolescent males in clinical models. It focuses on identifying and analyzing professional perceptions of their health demands. Adolescence is a period of exploration and self-discovery, crucial for the development of gender identity and sexual orientation
Barriers to access to health services for women and children in Latin America	Houghton et al. [14]	2022	Argentina	Cross-sectional descriptive study	The study analyzed barriers to accessing health services for women and children in Latin America. It identified healthcare infrastructure, implementing gender equity and empowerment policies, and educational campaigns to change social norms. These solutions aim to increase the use of essential health services and reduce access inequalities
Social Determinants of Access to Health Services for Children with Disabilities	Hurtado et al. [15]	2018	Colombia	Cross-sectional descriptive study	The social determinants of access to health services for children with physical disabilities in Buenaventura, Colombia, were analyzed. Barriers identified included the lack of installed capacity, availability of services and promotion, as well as administrative, geographical, and mobility obstacles. The study proposed improving healthcare infrastructure and implementing intersectoral policies to address these limitations
Expressions of inequalities in access to health services in Latin America: a scoping review	Oliveira [9]	2024	Brazil	Scoping review	Inequalities in access to health services in Latin America over the past 10 years were reviewed, analyzing 272 articles. Socioeconomic, sociocultural, and geographical barriers limiting equitable access were identified. The study proposed improving healthcare infrastructure, implementing inclusive policies, and strengthening community education to reduce these inequalities and improve health equity
Integrated Management of Childhood Illnesses implementation-related factors at 18 Colombian cities	García Sierra et al. [16]	2020	Colombia	Cross-sectional descriptive study	The implementation of the Integrated Management of Childhood Illness (IMCI) strategy in 18 Colombian cities was evaluated. Barriers identified included lack of essential medicines, necessary supplies, and second-level care. The study proposed improving resource availability and integrating the health system to reduce fragmentation and improve child care
Knowledge, access and use of the health system by migrant adolescents in Chile: Results of an exploratory study	Obach et al. [17]	2020	Chile	Qualitative study	The research evaluated the knowledge, access, and use of the health system by migrant adolescents in Santiago, Chile. Barriers identified included migration status, the perceived need for adult accompaniment, and experiences of discrimination. The study proposed improving information about the health system, facilitating access to sexual and mental health services, and strengthening the health sector’s presence in schools
Barriers due to specific diseases (***n*** = 10)					
Accessibility in Health: Review on Children with Disabilities in Brazil-Peru-Colombia	Dos Santos et al. [18]	2019	Brazil	Systematic review	20% of disabilities are congenital. In Latin America, the lack of health services severely affects children with functional diversity. Barriers include motor difficulties, family vulnerability, scarcity of specialized services, the lack of information, late diagnoses, and inadequate public policies and infrastructure
Assessment of Access to Primary Health Care among Children and Adolescents Hospitalized Due to Avoidable Conditions	Ferrer et al. [19]	2016	Brazil	Cross-sectional descriptive study	The study evaluates access to Primary Health Care (PHC) in children and adolescents hospitalized for avoidable conditions. Results show that 65.2% of hospitalizations were for PHC-sensitive conditions. Barriers include inadequate access, preference for emergency services, and professional attitudes that reinforce inappropriate ideas about service use
Challenges in the Provision of Pediatric Palliative Care in Mexico: A Cross-SectionalWeb-Based Survey	Grüneberg et al. [20]	2024	Mexico	Cross-sectional descriptive study	Research evaluating the challenges in providing pediatric palliative care in Mexico through an online cross-sectional survey. Barriers identified include the lack of home support teams, the absence of training centers, and legal and economic protection for parents. The study proposed increasing awareness and improving the education and training of health professionals
Determinants of care seeking for children with pneumonia and diarrhea in Guatemala: implications for intervention strategies	Bruce et al. [21]	2014	Guatemala	Qualitative study	The study analyzed the determinants of care seeking for children with pneumonia and diarrhea in Guatemala. Barriers identified included the distance to health centers, the lack of knowledge about danger signs, and perception of disease severity. The study proposed improving community education, increasing accessibility to health services, and strengthening community emergency plans
Effects of the COVID-19 Pandemic on Social Determinants of Families in the Metropolitan Area of Bucaramanga	Niederbacher et al. [22]	2023	Colombia	Cross-sectional descriptive study	Research focused on the effects of the pandemic on the social determinants of families in Bucaramanga. Barriers identified included the cancellation of medical appointments, mental health issues, and difficulties accessing virtual educational tools. The study proposed maintaining essential health services and educational programs during health emergencies to mitigate these impacts
Challenges, priorities, barriers to care, and stigma in families of people with autism: Similarities and differences among six Latin American countries	Paula et al. [23]	2020	Colombia, Argentina, Uruguay, Brazil, Mexico	Qualitative study	The study analyzed challenges, priorities, barriers to care, and stigma in families of people with autism in six Latin American countries. Barriers identified included long waiting lists, high treatment costs, and the lack of specialized services. The study proposed increasing community awareness, improving education, and strengthening specialized services to address these limitations
Healthcare Disparities in Atopic Dermatitis in Latin America: A Narrative Review	Sanchez et al. [24]	2022	Colombia, Argentina, Uruguay, Brasil, Mexico	Narrative review	This review highlights disparities in access to care for atopic dermatitis in Latin America. Barriers identified included insufficient knowledge of the disease, cultural and linguistic barriers, stigmatization, unequal distribution of resources, and lack of local clinical guidelines. The study proposed improving education, telemedicine, multidisciplinary teams, and locally adapted clinical guidelines
Health status and barriers in health care for children with birth defects born between 2011 and 2017 in two institutions in Cali	Imbachi et al. [25]	2020	Colombia	Retrospective study	A retrospective study evaluating the implementation of the Integrated Management of Childhood Illness (IMCI) strategy in 18 Colombian cities. Barriers identified included the lack of essential medicines, necessary supplies, and second-level care. The study proposed improving resource availability and integrating the health system to reduce fragmentation and improve child care
Meeting of the Pediatric Cancer Working Group (Washington, D.C. February 2–3, 2017)	Luciani et al. [26]	2017	PAHO Member Countries	Expert consensus	The meeting of the Pediatric Cancer Working Group in Washington, D.C. (February 2–3, 2017) addressed barriers to early diagnosis and effective treatment of pediatric cancer in Latin America and the Caribbean. Barriers identified included limitations in access to health services, funding, and international collaboration, highlighting the need for sustainable strategies to improve care and reduce inequalities
The Mental Health Care Gap among Children and Adolescents: Data from an epidemiological survey from Four Brazilian Regions	Paula et al. [27]	2014	Brazil	Cross-sectional descriptive study	The study on the mental healthcare gap among children and adolescents in four Brazilian regions revealed that only 19.8% of children with psychiatric disorders received care in the past 12 months. Barriers identified included structural, psychosocial, and demographic factors such as gender, school performance, and socioeconomic status
Vaccination barriers (*n*=5)					
Barriers and facilitators to vaccination in Latin America: a thematic synthesis of qualitative studies	Roberti et al. [28]	2024	Argentina	Qualitative study	This research aims to analyze barriers and facilitators of vaccination in Latin America through a thematic synthesis of qualitative studies. Facilitators include the perception of vaccination as an effective strategy and recommendations from healthcare professionals. Barriers encompass the lack of information, structural issues, religious beliefs, and safety concerns
Racial inequalities in child vaccination and barriers to vaccination in Brazil among live births in 2017 and 2018: an analysis of a retrospective cohort of the first two years of life	Boing et al. [29]	2024	Brazil	Cohorts study	The study analyzed racial inequalities in childhood vaccination in Brazil between 2017 and 2018. It identified significant barriers for Black mothers, such as difficulties accessing vaccination centers. The proposed strategies include equitable public policies and improving healthcare services to address these inequalities and ensure timely vaccination
Social considerations affecting acceptance of HPV vaccination in Colombia. A systematic review	Palencia-Sánchez et al. [30]	2020	Colombia	Systematic review	The study reviewed social factors affecting the acceptance of the HPV vaccine in Colombia. It proposed improving education about HPV, strengthening communication between healthcare professionals and the community, and using media as facilitators. It also recommended including the vaccine in insurance plans to improve access
Vaccination coverage of triple viral and poliomyelitis in Brazil, 2011–2021: temporal trend and spatial dependency	Palmieri et al. [31]	2023	Brazil	Ecological study	A retrospective study evaluated the coverage of the MMR (measles, mumps, and rubella) and polio vaccines in Brazil between 2011 and 2021, highlighting a decrease in coverage. It proposed strategies such as awareness campaigns, improving healthcare infrastructure, and policies to increase confidence in vaccines. These solutions aim to address regional disparities and improve vaccination coverage
Vaccination coverage, barriers and vaccine hesitancy in children up to 24 months old: a population survey in a state capital in the Western Amazon	Macedo et al. [32]	2024	Brasil	Cross-sectional descriptive study	Research that evaluated vaccination coverage, barriers, and hesitancy in children under 24 months in Rio Branco, Acre. It proposed improving vaccine availability, training healthcare professionals, and educational campaigns to combat misinformation. The results showed coverage below 80%, with the meningococcal C vaccine being the least administered on time
Strategies (*n*= 5)					
Health system approaches are needed to expand telemedicine use across nine latin american nations	LeRouge et al. [33]	2019	Argentina, Chile, Colombia, Costa Rica, Guatemala, Mexico, Panamá, Perú, Uruguay	Cross-sectional descriptive study	The study on telemedicine in Latin America examines how the COVID-19 pandemic accelerated the adoption of telemedicine services in the region. Regulatory, legal, and technological barriers are identified, and strategies are proposed to improve infrastructure and training. The results highlight the need for public policies that promote the integration of telemedicine into healthcare systems
Impact of a teaching hospital-based multidisciplinary telemedicine programme in Southwestern Colombia: a cross-sectional resource analysis	Prada et al. [34]	2024	Colombia	Cross-sectional descriptive study	A study on the telemedicine program “TeleSalud” at a high-complexity hospital in Cali, Colombia, identified key strategies: improving technological infrastructure, training medical staff in digital tools, and establishing clear policies to integrate telemedicine into the healthcare system. These strategies aim to overcome geographical and socioeconomic barriers, optimizing medical care
Use of Web-based Parent-adolescent Health Promotion Program among Puerto Ricans	Villarruel et al. [35]	2018	Puerto Rico	Experimental study	The study evaluated the web program “Cuídalos” to promote health among Puerto Rican parents and adolescents. It proposed improving access to computers and the Internet, and training parents in the use of digital tools. The results showed low participation, highlighting the need for strategies to increase the use of digital health resources and reduce access disparities
Telemedicine: Pediatric Applications	Burke Jr et al. [36]	2015	USA	Narrative review	The research analyzes the use of telemedicine in pediatrics, focusing on children with chronic diseases, those requiring postoperative follow-up, palliative care, and patients from remote areas. It proposes improving technological infrastructure, training healthcare professionals, and establishing appropriate reimbursement policies. The results show an improvement in care and access for these populations
Assessing the use of cell phones to monitor health and nutrition interventions: Evidence from rural Guatemal	Ceballos et al. [37]	2020	Guatemala	Experimental study	This study evaluated the use of mobile phones to monitor health and nutrition interventions in rural Guatemala. It proposed improving mobile phone penetration, sending SMS reminders, and making phone calls to increase response rates. The results showed that phone calls are more effective than SMS, suggesting their use for efficient and real-time data collection

**Table 3:** Result synthesis. Own elaboration

Barrier category	Results
Sociodemographic barriers	• 28.57% of the articles focused on Sociodemographic barriers. • The most frequently described barrier was difficulty obtaining money for the healthcare access or treatment, followed by the difficult access to the healthcare facility [34] • Geographical distance and the lack of resources as a cause of difficulty in accessing timely and adequate health services [7, 12, 16, 33]. • It was shown that many families are willing to travel in search of free healthcare services [11] • The main factors conditioning the occurrence of barriers in healthcare were income (44%), schooling level (35%), the limits to social mobility and transportation (13.7%), and housing conditions (7.4%) [9, 16] • For migrants, one of the barriers to accessing healthcare was registering in the health system [17].
Barriers due to specific diseases	• 35.7% of the articles focused barriers due to specific diseases • Theres a big complexity of access and accessibility to health services for children with various disabilities, where 7.14% of total articles talked about this disadvantage; that’s why the results point to planning strategies to improve healthcare for these children [18] • One of the articles proceed with a survey, where 91% of respondents replied that they take the child to the Primary Health Care Service when a routine checkup, but only 24% takes them when there is a new health problem [19] • It was noted that most of the diseases were connected to specific barriers, most households were located 5 km from the main town (57.7%) [20]
Vaccination barriers	• 17.86% of the articles focused on vaccination barriers • Vaccination was widely recognized as an effective strategy to prevent contagious diseases; however, the results showed the lack of guidance on vaccination and post-vaccination, safety concerns, misconceptions, and stigma specially in sexually transmitted diseases; also, the results showed that from the perspective of users, the main barriers to vaccinations were vaccine shortages at the health facility this linked to financial problems. In various countries of Latin America, fear of crime and social violence cited as a barrier to vaccination as it limited access to health facilities undermined the efforts [28–32] • Several studies highlight vaccination strategies as facilitators to prevent diseases. However, Roberti et al. emphasized the lack of information or knowledge in rural areas about the necessary vaccines for each stage of life as a significant barrier in the pediatric population [28]
Strategies	• 17.86% of the articles focused on strategies that target the barriers to accessing pediatric healthcare in Latin America • Even when strategies were made, for example, the web-based parent-adolescent health promotion program, parents stumble upon barriers like not easy access to computers (*n* = 134,48.6%) or did not know how to access to the program (*n* = 56, 20.3%) [15] • In this article, a survey was made to nine countries to see the use of telemedicine in the health system, an average of 65% of the hospitals in each country responded to the survey regarding the use of telemedicine. The use of ranged was 25% in the hospital in Colombia and 65% of the hospitals in Chile [28] • The information presented above is due to the lack of training in telemedicine platforms for both healthcare professionals and patients, ensuring they are prepared by integrating these tools into their daily practice [33]

## Results

**Fig. 2:**
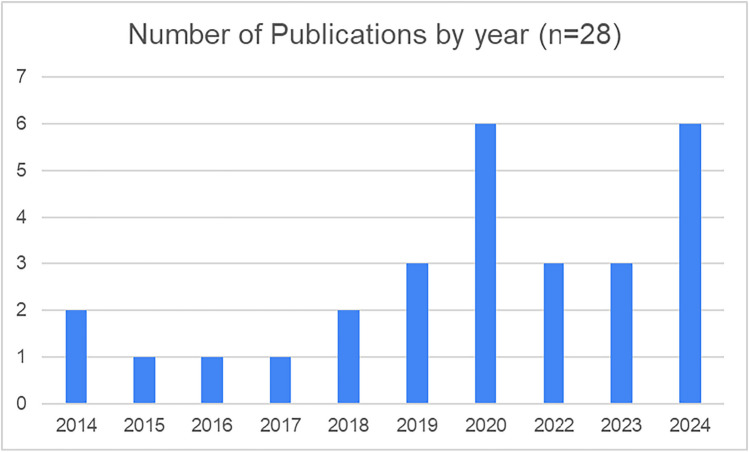
Number of publications by year

From the three databases consulted, 1538 articles were identified, of which 36 of which were duplicates, leaving a total of 1502 manuscripts. During the title and abstract screening, 58 articles were preliminarily identified as meeting the selection criteria. The remaining articles did not address topics related to barriers to healthcare access in the pediatric population or focused on other themes unrelated to the study’s objective. Finally, in the full-text screening, it was determined that 28 articles met the selection criteria and answered the exploratory question of the study. The main reason for discarding articles was the inability to access them (*n* = 15). The most common type of study was cross-sectional descriptive (*n* = 12, 41.38%), followed by qualitative studies (*n* = 5, 17.24%) and systematic reviews (*n* = 4, 13.33%). Other methodologies found included narrative reviews (*n* = 2, 6.67%), experimental studies (*n* = 2, 6.67%), cohort studies (*n* = 2, 6.67%), exploratory systematic reviews (*n* = 1, 3.33%), ecological studies (*n* = 1, 3.33%), and expert consensus (*n* = 1, 3.33%). Additionally, the year with the highest number of publications was 2024 (*n* = 7, 23%), followed by 2020 (*n* = 6, 20%). The rest of the publications by year can be seen in Fig. 2.

The articles were classified into categories. The first was the identification of sociodemographic barriers. Eight studies addressed these barriers, highlighting the costs associated with healthcare, the ability to pay, and the distance to health centers. In 25% of these articles, critical factors including the location and infrastructure of the centers are associated with transportation difficulties for families [7, 16]. These articles highlight the importance of implementing comprehensive policies and adapting them to each country’s specific sociodemographic challenges [7, 12, 16].

Another category included vaccination barriers. Six articles highlighted the importance of vaccination in pediatric patients (*n* = 6,17,86%). Immunization plays a fundamental role in preventing infectious diseases and reducing child mortality [29]. Several studies highlight vaccination strategies as facilitators to prevent diseases, but the lack of information or knowledge in rural areas about the necessary vaccines for each stage of life is a significant barrier in the pediatric population [28]. Similarly, Palmieri et al. agree that the lack of vaccine availability, unfavorable beliefs, and misinformation on the subject, especially in rural areas, negatively influence parents’ decisions to vaccinate their children [31]. 33.3% of the articles expressed doubts about vaccine safety and insufficient and confusing information about the vaccines [28, 31].

During the research, articles highlighted specific pathologies were also found, and the most relevant ones were selected to represent some of them. Ten articles highlighted the barriers linked to specific prevalent diseases (*n* = 10, 35.71%), in which they studied the access to primary healthcare for children and adolescents hospitalized for acute conditions and preventable diseases such as pneumonia and diarrhea care in children from rural areas in Guatemala and other countries in Latin America, revealing significant barriers in both contexts [19, 21]. In Brazil, external barriers associated with hospitalizations that could have been treated in primary care were identified [19]. In Guatemala, the search for formal care for pneumonia and diarrhea was limited, influenced by factors such as lack of emergency plans, perception of disease severity, and distance to health centers [21]. Additionally, pathologies related to birth, such as congenital diseases and disabilities, were analyzed [18, 25]. Studies have identified some of the gaps in accessing healthcare services in these populations from their vulnerability, highlighting external barriers associated with the Latin American context and its public policies, as well as those related to disability and its associated comorbidities [18, 25].

Regarding strategies to mitigate healthcare access barriers for the Latin American pediatric population, telemedicine has been a key tool that has gained strength in recent years. Additionally, the implementation of national policies that support and regulate the use of telemedicine requires improving technological infrastructure, especially in rural and underserved areas, to ensure adequate connectivity for patients [34, 38]. Furthermore, the importance of providing continuous training to healthcare professionals in the use of telemedicine technologies is highlighted, ensuring they are prepared to integrate these tools into their daily practice [33]. Ensuring funding and resources for its implementation guarantees the sustainability of telemedicine programs [33]. Finally, the adoption of a comprehensive approach that addresses regulatory, legal, financial, technological, organizational, and human barriers in a coordinated manner is emphasized to maximize the impact of telemedicine and improve access to high-quality healthcare services in the region [34].

## Discussion

This systematic review provides an analysis of the barriers to healthcare access in rural pediatric populations in Latin America, focusing on the various strategies that have been used, which have been effective in improving healthcare access for this population. It identifies gaps and limitations that are more commonly found in this population, providing recommendations that may help reach a consensus in the future to improve pediatric health.

### Sociodemographic Barriers in Pediatric Healthcare

It is estimated that around 200 million children under the age of 5 living in low- and middle-income countries do not reach their potential due to exposure to environmental, biological, and psychosocial risk factors [11]. Several authors agree that geographical barriers hinder children’s access to healthcare services, as long distances and insufficient transportation prevent them from reaching health centers [7, 11, 12, 16]. This implies that healthcare becomes fragmented, and continuity of care is not maintained, leading parents to take their children to health centers only when they are sick, preventing the proper implementation of health promotion and prevention strategies. Ultimately, this represents a higher risk of developing short- and long-term health complications in children and higher costs for healthcare systems. On the other hand, although the region has a high rate of affiliation with social security systems, the lack of economic resources, excessive bureaucracy, and prolonged response times, combined with the long distances families must travel, further hinder timely healthcare access. Therefore, governments should implement strategies and policies that ensure more equitable access to healthcare services [12, 24].

### Vaccination Barriers

Latin America continues to have low vaccination coverage, which represents a significant public health problem. To achieve broad vaccination coverage, Robierti et al. identify facilitators in vaccination access and some barriers, highlighting the lack of truthful and understandable information, influenced by cultural and religious factors, which makes families and caregivers reluctant to seek this service [11]. Additionally, the inability of many families and healthcare systems to meet this need, combined with infrastructure incapable of properly storing these medications, reduces the rates of complete vaccination in children. However, other racial barriers have been identified. Boing et al. found that being of African descent meant that mothers and their children were less likely to have access to vaccination compared to Caucasian mothers and children. It is imperative that governments implement strategies to combat systemic racism in healthcare systems [29]. Communities also play an important role in the successful implementation of vaccination programs. An example of this was the stance of a community in Colombia, where misinformation provided by some media outlets made it difficult for parents to accept vaccinating their daughters with the human papillomavirus (HPV) vaccine [14]. This highlights the importance of implementing appropriate information campaigns and the impact that communities can have on children’s health decisions.

### Barriers Due to Specific Prevalent Diseases in the Pediatric Population

In the region, several studies have analyzed the barriers to healthcare access faced by children with certain medical conditions. While some of these barriers are specific to each disease, many authors agree on the presence of some common barriers for different types of diseases. Firstly, both medical personnel and caregivers have identified a lack of institutional and governmental support due to insufficient human resources or infrastructure to meet the special needs of this population [23], as evidenced by studies conducted on pediatric populations with mental health disorders and cancer [23, 33, 34]. Furthermore, the education of both parents and healthcare professionals is crucial to reducing the healthcare gap for these children and adolescents. On the one hand, it is important for parents and caregivers to be trained to recognize the warning signs of their children’s diseases [21]; this means they must understand what the disease is, how it manifests, and how it is treated. To achieve this, healthcare professionals must be prepared and have sufficient knowledge to provide adequate care to children with special health needs and to clarify any doubts caregivers may have. This could be achieved by promoting the creation of new scientific knowledge through local clinical practice guidelines [24, 27]. Additionally, considering that Latin America is a culturally and ethnically diverse region, the training of healthcare personnel should not be limited to the clinical aspect alone. It is relevant for healthcare personnel to acquire new knowledge to approach different ethnic contexts more appropriately and thus achieve a greater impact [24, 27].

Regarding prevalent pediatric pathologies, articles of significant clinical importance related to pediatric palliative care in Mexico and pediatric cancer management in Latin America were found. In Mexico, the main barriers identified by professionals in care units were the lack of equipment and support networks outside the hospital; regarding the centers, the absence of training centers and continuous education in palliative care was identified [20]. The meeting highlighted the high mortality from pediatric cancer in the region of countries associated with PAHO [6]. Both studies mention governmental and civil obstacles, highlighting the lack of public policies, lack of unification among governments, civil society, and academic institutions. Additionally, the lack of early diagnostic identification associated with administrative gaps and problems in specialized care was highlighted.

Furthermore, studies on the disparity in mental healthcare for children and adolescents in Brazil and the challenges faced by caregivers of pediatric populations with autism in six Latin American countries were analyzed, examining the most significant obstacles to comprehensive care for these pathologies. In Brazil, it was identified that only 19.8% of the pediatric population with psychiatric disorders consulted mental health services in the last year, with gender, school performance, and socioeconomic level being the main factors associated with lack of access [27]. In Latin America, caregivers of children with autism revealed that administrative barriers such as waiting lists, treatment costs, the lack of specialized services, and psychosocial barriers such as high levels of stigmatization and discrimination were among the obstacles to obtaining comprehensive care [23]. Both studies highlight the gaps in access to specialized mental health services, their follow-up, and the need for strategies to reduce inequalities and stigma in Latin America.

Among skin conditions, atopic dermatitis was noted as a neglected pathology in Latin America due to clinical obstacles such as insufficient knowledge about the disease when diagnosing it, the lack of local clinical guidelines, complex patient pathways, and limited consultation time; sociodemographic obstacles included cultural and linguistic barriers and patient stigmatization; and administrative obstacles included unequal resource distribution [24].

### Strategies to Overcome Healthcare Access Barriers in the Latin American Pediatric Population

There are strategies that have been implemented to improve healthcare access in Latin America. Since the COVID-19 pandemic, strategies that remain relevant due to their usefulness in addressing geographical, environmental, and economic barriers, such as telemedicine, have been implemented [34]. LeRouge et al. have presented this tool as a response to the problem of inequality in access to medical consultations and how it has increased the efficiency of healthcare for non-urgent reasons in primary stages, reducing the number of complications due to the lack of care. In the application of this tool in remote communities that have access only by air or sea, several shortcomings were identified to ensure this technology, such as the use of a stable internet network to ensure continuous connectivity [38]. Additionally, several authors have identified other areas where these technologies could be further developed, such as expanding modalities in tele-education, teleconsultation, telepractice, and tele-research, using digital applications to strengthen remote and comprehensive healthcare access for all [9].

### Study Limitations

This scoping review is based on a limited number of articles, which may not fully represent the diversity of healthcare access barriers for children and adolescents in rural Latin American regions. One of the limitations encounters were that 33% of the items that met the criteria were not retrieved due to a combination of technical and content-related factors. Some of the articles were behind paywalls or restricted access platforms and others did not align with the proposed research question leading to the exclusion during the selection process. An important limitation is the variability in the quality and focus of the studies. Some articles provide robust quantitative data on the magnitude of the identified barriers, while others rely on narratives and qualitative observations. This methodological heterogeneity makes it difficult to compare and synthesize the findings. Therefore, to extrapolate the findings to real-life situations, experimental studies with significant samples and ideally from culturally and ethnically diverse populations are required to achieve results with greater reach and provide more accurate data.

Moreover, most studies focus on sociodemographic and vaccination barriers, leaving other barriers related to specific diseases and strategies to mitigate these barriers underexplored. For example, Shibukawa et al. [12] identified significant barriers such as geographical distance and lack of economic resources. However, the review does not delve into how these barriers vary between different subregions or specific populations in Latin America. This could limit the applicability of the recommendations to diverse local contexts.

## Conclusions

This scoping review identifies various barriers affecting healthcare access for the pediatric population in Latin America, allowing them to be grouped into categories such as sociodemographic barriers, vaccination barriers, barriers related to specific diseases, and strategies to mitigate them [33]. It also highlights telemedicine as a key strategy to improve access in remote areas, allowing families to consult with healthcare professionals specialized in pediatric care, ensuring timely and effective interventions for children. However, its effectiveness depends on internet availability, adequately trained healthcare personnel, and the necessary infrastructure to provide this type of care [24].

In conclusion, it is also important to acknowledge that while this study provides valuable information, it also has limitations, due to the restricted number of reviewed articles and the variability in the quality and focus of the included studies. It is important to implement technological strategies, and significant samples are required to ensure the applicability of the recommendations [27].

## Data Availability

Not applicable.
